# Adsorption of Zn^2+^ from Synthetic Wastewater Using Dried Watermelon Rind (D-WMR): An Overview of Nonlinear and Linear Regression and Error Analysis

**DOI:** 10.3390/molecules26206176

**Published:** 2021-10-13

**Authors:** Wahid Ali Hamood Altowayti, Norzila Othman, Adel Al-Gheethi, Nur Hasniza binti Mohd Dzahir, Syazwani Mohd Asharuddin, Abdullah Faisal Alshalif, Ibrahim Mohammed Nasser, Husnul Azan Tajarudin, Faris Ali Hamood AL-Towayti

**Affiliations:** 1 Micro-Pollutant Research Centre (MPRC), Department of Civil Engineering, Faculty of Civil Engineering and Built Environment, Universiti Tun Hussein Onn Malaysia, Parit Raja 86400, Johor, Malaysia; adel@uthm.edu.my (A.A.-G.); hasniza_dzahir@yahoo.com (N.H.b.M.D.); syazwani.asharuddin@gmail.com (S.M.A.); 2 Jamilus Research Centre for Sustainable Construction (JRC-SC), Faculty of Civil Engineering and Built Environment, Universiti Tun Hussein Onn Malaysia, Parit Raja 86400, Johor, Malaysia; faisalalshalif@gmail.com (A.F.A.); alhawry5@gmail.com (I.M.N.); 3 Division of Bioprocess, School of Industrial Technology, Universiti Sains Malaysia, Gelugor 11800, Pulau Pinang, Malaysia; 4 Departement of Civil and Environmental Engineering, Universiti Teknologi PETRONAS, Seri Iskandar 32610, Perak, Malaysia; Engaltowayti@gmail.com

**Keywords:** adsorption, Zn^2+^, watermelon rind, isotherm, kinetics

## Abstract

Sustainable wastewater treatment is one of the biggest issues of the 21st century. Metals such as Zn^2+^ have been released into the environment due to rapid industrial development. In this study, dried watermelon rind (D-WMR) is used as a low-cost adsorption material to assess natural adsorbents’ ability to remove Zn^2+^ from synthetic wastewater. D-WMR was characterized using scanning electron microscope (SEM) and X-ray fluorescence (XRF). According to the results of the analysis, the D-WMR has two colours, white and black, and a significant concentration of mesoporous silica (83.70%). Moreover, after three hours of contact time in a synthetic solution with 400 mg/L Zn^2+^ concentration at pH 8 and 30 to 40 °C, the highest adsorption capacity of Zn^2+^ onto 1.5 g D-WMR adsorbent dose with 150 μm particle size was 25 mg/g. The experimental equilibrium data of Zn^2+^ onto D-WMR was utilized to compare nonlinear and linear isotherm and kinetics models for parameter determination. The best models for fitting equilibrium data were nonlinear Langmuir and pseudo-second models with lower error functions. Consequently, the potential use of D-WMR as a natural adsorbent for Zn^2+^ removal was highlighted, and error analysis indicated that nonlinear models best explain the adsorption data.

## 1. Introduction

Wastewater treatment is one of the most important topics of scientific study, covering a wide range of disciplines. Over the previous three decades, wastewater treatment for heavy metal removal has been a focus of study [1]. Worldwide, as industrialization continues to grow, the water system has been badly impacted by pollution [2]. Industrial effluents have a negative impact on water ecosystems and living organisms [3]. Heavy metals such as arsenic, copper, and zinc are found in the effluent from industries such as fertilizer, mining, refining, pulp and paper, textile, cement, etc. Zn^2+^ is a vital nutrient for humans. In addition, it controls a variety of biological activities. But too much Zn^2+^ may lead to a series of health issues, including stomach pains, skin irritations, nausea and anaemia [4]. Almost all of these municipal and industrial effluents are dumped directly into bodies of water without adequate treatment, and as a result, they become severely contaminated [5,6]. Aquatic creatures are also killed as a result of the water’s increasing toxicity over time. Since certain heavy metals like Zn^2+^ do not biodegrade, they pose a serious threat to the environment and tend to accumulate in living creatures, leading to illnesses and diseases. As a result of bio-magnification, their concentration rises over time at every level of the food chain, and they progress to higher levels. The water becomes unfit for human consumption as a result of pollution. Aquatic life is threatened by even a relatively tiny concentration of heavy metals in water systems [7,8,9]. This means that in order for the water ecosystem to thrive, heavy metals such as Zn^2+^ must be removed and reduced from wastewater.

Heavy metals such as Zn^2+^ can be removed by chemical precipitation, ion exchange, adsorption, membrane filtration, and electrochemical treatment methods [10]. Researchers have used microbe biomass as an adsorbent to successfully remove heavy metals from wastewater [11,12,13]. Agriculture wastes and other adsorbents have been extensively investigated because of their local availability. It’s becoming increasingly common to produce bio-adsorbent from waste biomass because of its low cost and high effectiveness, especially in adsorption procedures to remove harmful chemicals from wastewater [14]. Surface chemistry and textural characteristics, such as surface area and porosity, describe mesoporous silica. Starting material and preparation procedures have a significant impact on these adsorbents. There are several waste biomaterials with high carbon content, including coconut shells [15], almond shells and walnut shells [16], rice stalk [17], pine sawdust [18], and orange peel [19] and watermelon rind [20]. Carotenoids and amino acids such as citrulline and other phytochemical substances may be found in the watermelon rind, which is often discarded in huge quantities and is made of carbonaceous components such as polysaccharides e.g., cellulose and pectin [21,22,23]. Mesoporous materials may be synthesized from the rind because of the presence of these chemicals in the rind. Toxic heavy metals may be bound to the rind of watermelon due to hydroxyl and carboxyl groups (cellulose and pectin) found there [24]. However, watermelon rind also contains a substantial quantity of phenolic chemicals and is capable of neutralising free radicals in the body (hydroxyl radical scavenger) [25]. Chemical activation has also produced natural adsrobents with a large surface area, which are widely utilised to remove contaminants [14,18,26,27,28,29,30].

To find the best adsorbent, equilibrium correlation analysis must be used to predict adsorption behaviour under different experimental conditions. This equilibrium correlation is derived using equilibrium isotherms and kinetics models, which are used to construct the correlation. Basically, these isotherms show how the adsorbate interacts with the adsorbent’s surface, such as by monolayer or multilayer adsorption [31,32]. It is also crucial to conduct thermodynamic experiments in order to ascertain whether the adsorption is spontaneous. In addition, it gives information about the optimal temperature range for adsorption and the type of adsorbent and adsorbate at equilibrium [33,34].

A waste biomaterial, dried watermelon rind has not yet been assessed as a raw material for the chemical activation technique by nitric acid (HNO_3_) to produce a low-cost natural mesoporous adsorbent. Hence, the main objective of the present work was to evaluate the usefulness of waste watermelon rind as an abundant and accessible precursor as a mesoporous material to remove Zn^2+^ from synthetic wastewater. The adsorption process has been used to optimize a number of different factors, including the initial concentration of Zn^2+^, adsorbent dose, contact time, pH, and temperature. A thermodynamic analysis was carried out to identify the reaction nature of the adsorption phenomena, and nonlinear and linear models were used to select an acceptable isotherm and kinetics model for experimental data. The optimal model for equilibrium data was also determined by doing an error analysis using four distinct error functions.

## 2. Materials and Methods

### 2.1. Preparation of Glassware

All glass apparatus used in the laboratory for this study was cleaned using cleaning chemicals before being immersed in 15% nitric acid (HNO_3_). Nitric acid is well-known for its ability to eliminate any residue left on the apparatus, and it has no effect on laboratory analysis. The equipment was then washed with distilled water and dried in an oven in accordance with Standard Methods for the Examination of Water and Wastewater, 23rd Edition [35].

### 2.2. Preparation of Dried Watermelon Rind Powder (D-WMR)

**Figure 1:**
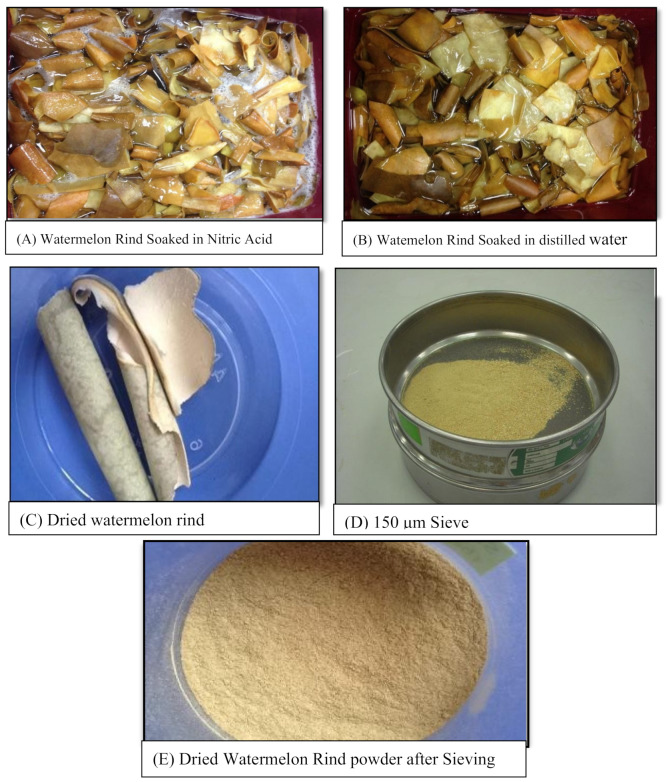
Steps for producing dried watermelon rind powder with 150 μm particle size.

Several stages are involved in the production of dried watermelon rind. First, the watermelon rind was washed and cleansed to eliminate any debris on the surface of the rind. The watermelon skin was then peeled to obtain the thinnest rind and sun-dried for two days. The watermelon skin was then immersed in 15% nitric acid (HNO_3_) for 24 h, as illustrated in Figure 1A. Nitric acid is utilized to activate adsorbents, resulting in a porous structure with a large surface area [14]. The watermelon rind was then rinsed and immersed in distilled water to eliminate any remaining nitric acid on the surface, as illustrated in Figure 1B. The watermelon rind is next baked in the oven at 60 °C until it is completely dry, as shown in Figure 1C. The grinder equipment used to powder the watermelon rind must be dry before usage. In the laboratory, the watermelon rind was sieved using a sieve size of 150 μm after being ground, as illustrated in Figure 1D. The powder of dried watermelon rind (D-WMR) (Figure 1E) was kept for future characterization and adsorption studies.

### 2.3. Characterization of Dried Watermelon Rind

X-ray fluorescence (XRF) and scanning electron microscopes (SEM) are used to analyze dried watermelon rind (D-WMR) powder. XRF was utilized to determine the chemical composition of the D-WMR, while a scanning electron microscope was employed to examine the microstructure of the D-WMR. In this experiment, the X-ray fluorescence (XRF) function is used to determine the elements in the D-WMR rind as a bioadsorbent and the proportion of each ion present in the sample. Dried watermelon rind powder samples (150 m in size) were placed in a palette and analyzed using XRF and SEM equipment. The scanning electron microscope (SEM) generates pictures with exceptionally high magnification and great resolution, and also has the capacity to generate localized chemical information. This implies that the equipment is a useful for resolving a wide variety of product and processing issues for a wide range of metals and materials. Consequently, the microstructure of the D-WMR was analyzed and confirmed using these devices.

### 2.4. Preparation of Synthetic Wastewater

The stock solution was diluted to obtain a standard solution and desirable concentration. Zinc stock solution (1000 mg/L) is made by adding 2.5 g zinc sulfate (ZnSO_4_) to 1 L deionized water. Synthetic wastewater is being diluted from the ZnSO_4_ stock solution. The following relationship may be used to calculate the required Zinc concentration:

### 2.5. Analysis of Zinc in Synthetic Wastewater

Atomic absorption spectroscopy (AAS) is the method used to analyze Zn^2+^ in synthetic effluent. AAS is a technique for determining the concentrations of chemical components in water samples by measuring the absorbed radiation. Zn^2+^ concentrations in synthetic wastewater were measured before and after the bioadsorption procedure.

### 2.6. Factors Affecting the Efficiency of Adsorption Process

Batch mode experiments were carried out in 250 mL conical flasks shaken with various pH (6–9), initial Zn^2+^ concentrations (100–600 mg/L), adsorbent dose (0.5–3 g/L), contact time (1–5 h), and temperature (20–50 °C) to assess optimal adsorption conditions. The absorption capacity of Zn^2+^ per gram biomass (mg/g) was determined using the following equation: [36,37]:

(1) $$q_{e}=\frac{C_{i}-C_{e}}{M}\;\times \;V$$

The Zn^2+^ removal percentage (R%) was determined using the concentration difference before and after adsorption as follows [38]:

(2) $$(R\%)=\frac{C_{i}-C_{e}}{C_{i}}\;\times \;100$$

where:

$C_{i}$: The initial concentration of Zn^2+^ ions in mg/L,

$C_{e}$: The final concentration of Zn^2+^ ions in mg/L,

M: The mass of WMR adsorbent (g),

V: The volume of the solution (L).

### 2.7. Thermodynamic, Isotherm and Kinetic Adsorption Models

The impact of various temperatures on the thermodynamic study of Zn^2+^ removal using dried watermelon rind (D-WMR) was investigated at various temperatures of 20, 30, 35, 40 °C (293.14, 303.15, 308.15 and 313.15 Kelvin). Atomic absorption spectroscopy (AAS) was used to examine the residual Zn^2+^ in the solution. The Langmuir and Freundlich nonlinear and linear models were used to evaluate the adsorption isotherm for Zn^2+^ using the optimum adsorbent (1.5 mg D-WMR) combined with various amounts of Zn^2+^ (100–600 ppm). The adsorption kinetic rate of Zn^2+^ was studied using pseudo-first-order and pseudo-second-order nonlinear and linear models throughout a range of incubation times (1–5 h).

### 2.8. Analytical Error Analysis

It is important to calculate the error function in order to identify the optimum fitting of nonlinear or linear models onto adsorption data [39]. A study of error functions is required to evaluate the models’ fit to experimental data. In most research, the coefficient of determination (R^2^) is likely the most commonly utilized function to determine the optimal isotherm and kinetics models [40,41]. Based on the magnitude of the coefficient of determination, the best-fit model is chosen. The model with the highest coefficient of determination value is thought to be the best match [42]. Furthermore, the less the numerical value of the error functions for a given model, the better the fit for that adsorption data. The formulae for the coefficient of determination and different error functions are as follows:

#### 2.8.1. Coefficient of Determination (R^2^)

In a regression model, the coefficient of determination (R^2^ or r-squared) is a statistical metric that indicates the proportion of variation in the dependent variable that can be explained by the independent variable. Moreover, the coefficient of determination indicates how well the data fits the model (the goodness of fit). The coefficient of determination can range from 0 to 1. Furthermore, the statistical measure is commonly stated as follows:

(3) $$R^{2}=1-\frac{\;\sum_{i=1}^{n}{\left(M_{t}-A_{t}\right)}^{2}}{\sum_{i=1}^{n}\left(A_{t}-A_{Ẍ}\right)}$$

#### 2.8.2. Mean Square Error (MSE)

Mean square error (MSE) is probably the most commonly used error metric. It penalizes larger errors because squaring larger numbers has a greater impact than squaring smaller numbers. The MSE is the sum of the squared errors divided by the number of observations as in the following:

(4) $$\mathrm{MSE}=\frac{1}{n}\sum_{i=1}^{n}\left(A_{t}-M_{t}\right)$$

#### 2.8.3. The Root Mean Square Error (RMSE)

The root mean square error (RMSE) Is the Square root of the Mean square error (MSE) and can be stated as the following equation:

(5) $$\mathrm{RMSE}=\sqrt{\frac{1}{n}\sum_{i=1}^{n}({A_{t}-M_{t})}^{2}}$$

#### 2.8.4. The Mean Absolute Deviation (MAD)

The mean absolute deviation (MAD) is the sum of absolute differences between the actual value and the forecast divided by the number of observations and can be expressed as the following equation:

(6) $$\mathrm{MAD}\;=\frac{\sum_{i=1}^{n}\left|A_{t}-M_{t}\right|}{n}$$

#### 2.8.5. Mean Absolute Percentage Error (MAPE)

Mean Absolute Percentage Error (MAPE) s the average of absolute errors divided by actual observation values and can be written as the following:

(7) $$\mathrm{MAPE}=\frac{\sum_{i=1}^{n}\left|\frac{A_{t}-M_{t}}{A_{t}}\right|}{n}$$

where:

$A_{t}$: Experiment data, $M_{t}$: Model date, $A_{Ẍ}$: Experiments average data,

n: number of experiments carried out

## 3. Results and Discussion:

### 3.1. Characterizations of Dried Watermelon Rind

#### 3.1.1. Physical Characterizations of Dried Watermelon Rind Using Scanning Electron Microscope (SEM)

**Figure 2:**
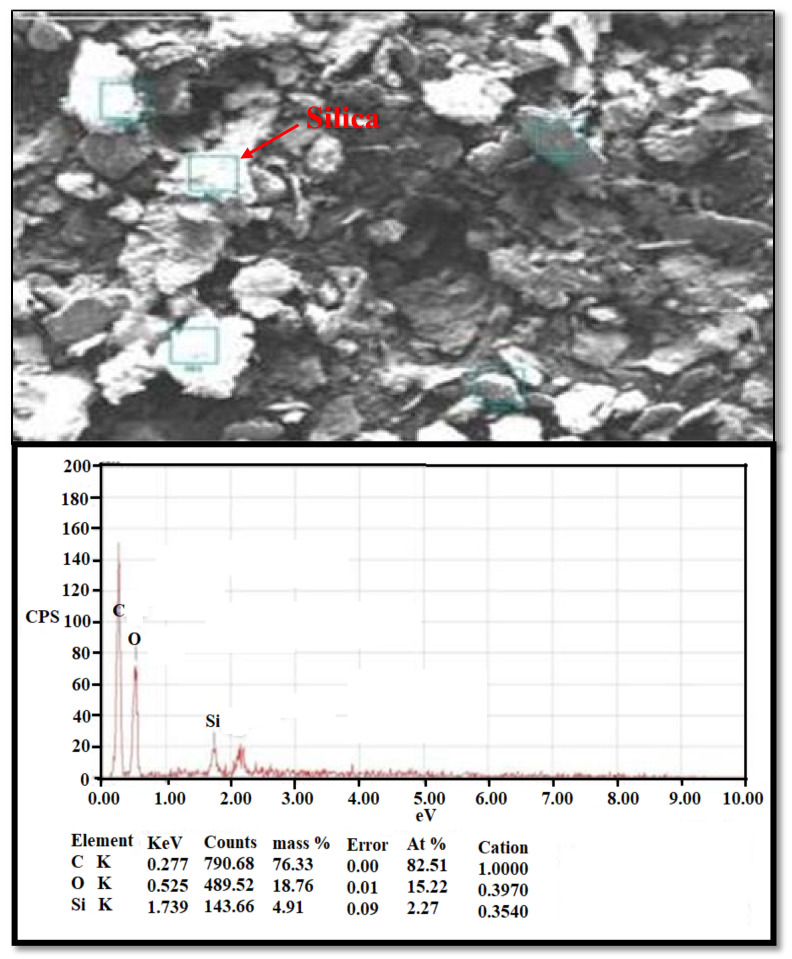
Scanning Electron Microscopic of watermelon rind analysis before adsorption of Zn^2+^ with EDX analysis.

**Figure 3:**
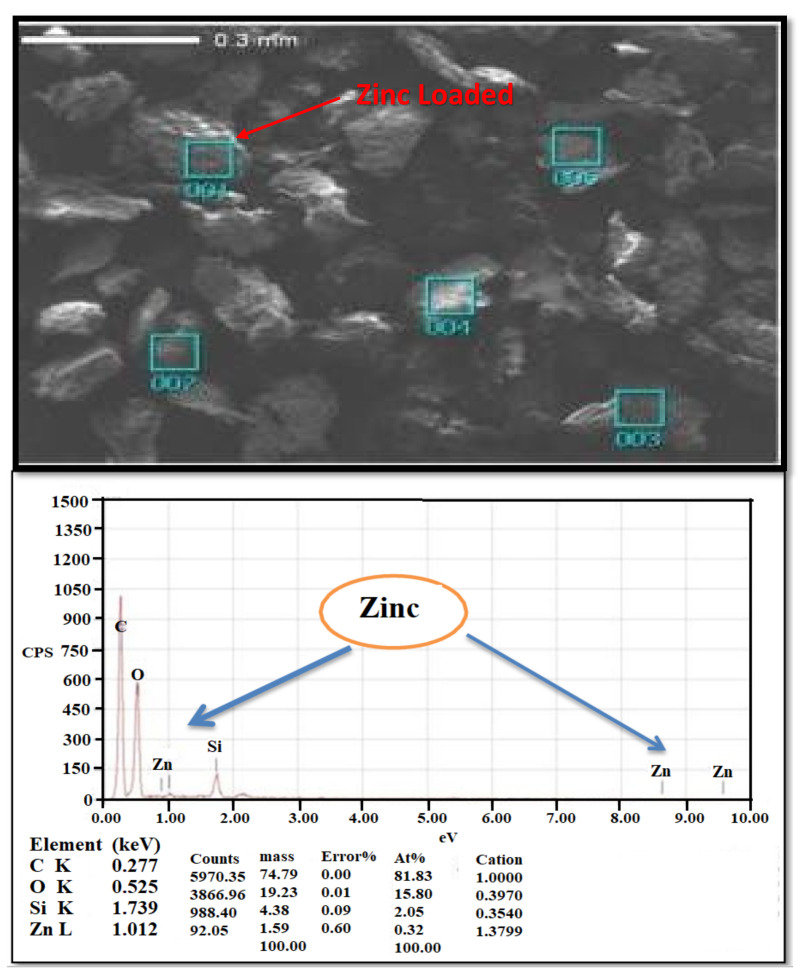
Scanning Electron Microscopic of watermelon rind after adsorption of Zn^2+^ with EDX analysis.

The microstructure and physical properties of watermelon rind powder are determined using a scanning electron microscope (SEM) before and after Zn^2+^ adsorption. SEM pictures of the microstructure of the watermelon rind adsorbent were used in this investigation. The microstructure is an important component in increasing percentage removal. The surface of the unloaded adsorbent (Figure 2) is rough, uneven, and porous, indicating that it has favourable properties for use as a natural adsorbent for the adsorption of metallic ions [43]. Furthermore, the loaded adsorbent’s SEM micrograph (Figure 3) reveals changes in shape and colour, indicating Zn^2+^ adsorption onto the D-WMR.

By generating X-rays owing to high-energy electrons, EDX analysis gives information on the elements. The watermelon skin has two hues, a white region and a black area, according to the EDX study in Figure 3. The white region represents mesoporous silica composition, but the black area represents a protein, which includes the most carbon and oxygen [44]. Figure 3 shows how the spectrum detects carbon, oxygen, and silicon in a dried watermelon rind, confirming the existence of mesoporous silica in dried watermelon rind.

#### 3.1.2. Chemical Composition of Dried Watermelon Rind Using X-ray Fluorescence (XRF)

X-ray Fluorescence (XRF) was used to examine the chemical composition of the D-WMR before and after the Zn^2+^ adsorption procedure. Table 1 presents a summary of the findings. Table 1 demonstrates that the proportion of heavy metals elements present in the D-WMR before the adsorption procedure is insufficient and may be ignored. Watermelon rind powder has a total of 83.70% SiO_2_, which is the greatest element content in dried watermelon rind, indicating the mesoporous structures of the watermelon rind. Furthermore, after the adsorption procedure, 24.40% of Zn^2+^ in dried watermelon rind (D-WMR) revealed that the Zn^2+^ ions were successfully removed from synthetic wastewater. The decrease in the percentage of silica content after zinc adsorption is due to silica’s capability in the ion exchange process over metals removal [45].

### 3.2. Factors Affection Adsorption Capacity

#### 3.2.1. Effect of pH against Zinc Removal

**Figure 4:**
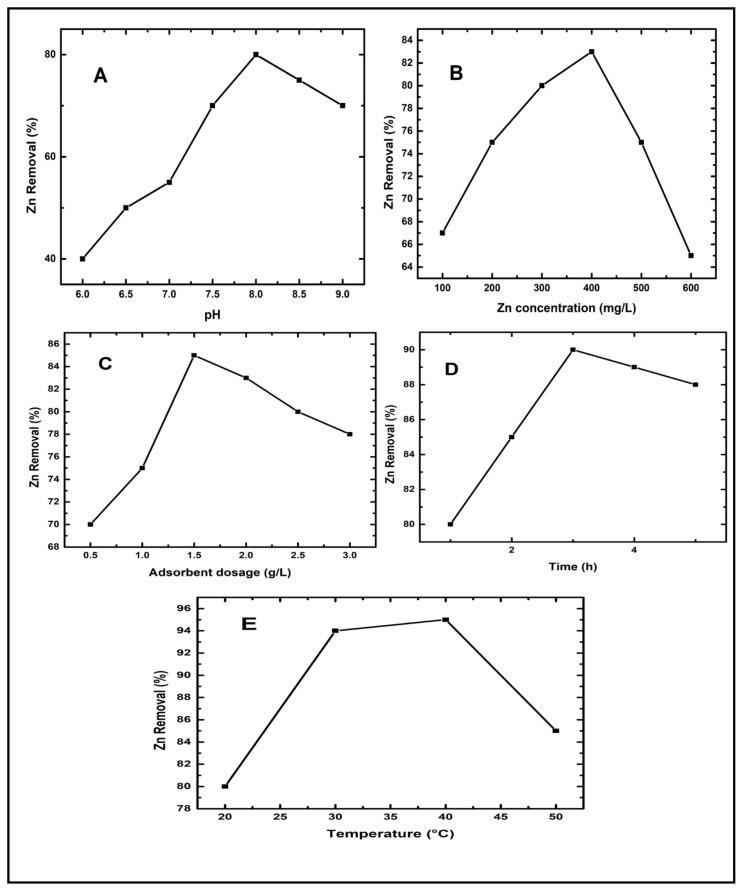
Effect of (**A**) pH, (**B**) Zn^2+^ ions concentration (mg/L), (**C**) D-WMR dosage (mg/L), (**D**) contact time (h) and (**E**) temperature (°C) for Zn^2+^ ions removal.

pH has been shown in several studies to have a substantial influence on heavy metal adsorption [31,32,34]. The adsorptive process is affected by changes in initial pH due to the dissociation of functional groups on the active sites on the D-WMR’s surface. The impact of pH on the removal of Zn^2+^ onto WMR from synthetic wastewater is shown in Figure 4A. It was observed that as the initial pH was raised, the removal efficiency of Zn^2+^ increased until pH was 8, but subsequently decreased beyond pH 8.0. D-WMR achieved a maximum removal of 80% at pH 8. At lower pH values, significant hydrogen ions compete for empty adsorption sites of adsorbents, which causes this phenomenon. The low efficiency in acidic solutions (pH less than 7) might be attributed to increased competition for adsorption sites between H^+^ and Zn^2+^ [46]. Adsorption increased when solution pH increased because additional metal-binding sites with negative charges could be exposed, attracting metal ions with positive charges and causing adsorption onto the adsorbent surface [47]. At pH values greater than 8.0, D-WMR’s elimination of Zn^2+^ decreased. There were more negative ions present when the pH was elevated above pH 8, and the Zn^2+^ was surrounded by anions. Zn^2+^ binding to adsorption sites on the surfaces of negative charge adsorbents is thus difficult due to the large concentration of hydroxyl ions in the water in alkaline pH [48].

#### 3.2.2. Effect of Zinc Concentration in Synthetic Wastewater against Zinc Removal

In the adsorption process, the initial concentration of adsorbate is crucial. Figure 4B depicts the elimination of Zn^2+^ from a solution employing watermelon rind powder as a natural adsorbent material at concentrations of 100, 200, 300, 400, 500, and 600 mg/L, respectively. The rate of adsorption was enhanced by increasing the initial Zn^2+^ concentration. This study used the optimal pH of the solution, as shown above, to get the best Zn^2+^ adsorption results. The percentage of adsorption increased from 67% to 83% when the initial concentration of Zn^2+^ was raised from 100 to 400 mg/L. According to the findings, the maximum removal was found to be 83%. Increased mobility can be due to an increase in adsorption capacity at high starting concentrations. Due to an increased potential difference and electrostatic adsorption, Zn^2+^ ions are more readily adsorbed on the D-WMR surface when the initial concentration is increased. However, a reduction in the effectiveness of Zn^2+^ ions removal was seen throughout the adsorption phase due to the occupancy of active sites. Zn^2+^ ions may be easily absorbed in low concentrations due to a large number of active contact sites on the adsorbent’s surface. Because more exchange sites are filled and saturated as Zn^2+^ ions concentrations grow, Zn^2+^ ions cannot be adsorbed. In this way, the rate of adsorption efficiency became constant over a period of time with the fixed adsorbent dosage of D-WMR. Because the driving force for adsorption is the initial concentration of Zn^2+^ ions, a high initial concentration of Zn^2+^ ions enhances the rate of adsorption. An increase in the concentration of Zn^2+^ ions had a substantial influence on the adsorption process at the start of the process, but as the process progressed, adsorption reduced [49,50].

#### 3.2.3. Effect of Adsorbent Dosage Concentration against Zinc Removal

For an initial Zn^2+^ concentration of 400 mg/L, the effect of the adsorbent dosage on Zn^2+^ removal was investigated. The adsorbent dosage was adjusted from 0.5 to 3 g/L, and the solutions were stirred for 3 h. The obtained findings demonstrate that the effectiveness of Zn^2+^ removal improves when the adsorbent dosage is increased (Figure 4C). At the optimal adsorbent dose of D-WMR (1.5 mg/L), the maximum Zn^2+^ removal was 85%. The increase in adsorbent dosage allows for more additional Zn^2+^ ion adsorption sites, which explains the improvement in efficiency [46]. Furthermore, with larger adsorbent concentrations, this rise is attributable to the greater availability of exchangeable sites or surface area. Therefore, if the adsorbent dose is increased, more surface area is accessible for adsorption, making Zn^2+^ ion penetration to the adsorption sites simpler. However, when equilibrium was reached, increasing the adsorbent dose had no impact [51]. Similar findings have been reported by a number of studies. According to Zhang, et al. [52], increasing the bentonite dose (0.2 to 2 g/L) enhances Zn^2+^ removal efficiency (100 mg/L). In addition, Mishra and Patel [53] reported the same effect when treating distilled synthetic water solutions containing 100 mg/L with kaolin (5 to 20 g/L).

#### 3.2.4. Effect of Contact Time against Zinc Removal

The impact of contact time on Zn^2+^ adsorption by D-WMR has been investigated. The effect of contact time was investigated across a time period of 1 to 5 h. In 100 mL of synthetic water, 1.5 g/L of D-WMR adsorbent was introduced to 400 mg/L of Zn^2+^, and Zn^2+^ removal was measured by atomic absorption spectroscopy. Figure 4D shows that increasing contact time increases Zn^2+^ ion removal efficiency until a threshold is reached when no more Zn^2+^ is removed. The removal percentage of Zn^2+^ ions was achieved during the first three hours, after which equilibrium was achieved. As a consequence, the improved removal efficiency was seen in the first three hours, but there was no substantial improvement in Zn^2+^ removal beyond that. As a result, during the optimum contact period (3 h), the maximum Zn^2+^ removal rate of 90% was attained. The Zn^2+^ removal will not increase with further contacting time since all binding sites have been saturated with a restricted adsorbent dose [54]. Similar findings were reported by Rana et al. [55], who discovered that increasing the contact period between the adsorbent and synthetic wastewater improved metal ion removal.

#### 3.2.5. Effect of Temperature against Zinc Removal

Temperature is an important element that has a significant impact on adsorption. Experiments were carried out at temperatures of 20, 30, 40, and 50 °C to investigate the temperature effect (Figure 4E). Adsorption of Zn^2+^ ions increased when the temperature was elevated from 20 to 40 °C, suggesting that the adsorption process was endothermic. At high temperatures, this might be attributed to activation and quicker migration of adsorbate toward the coordinating sites of adsorbent [56]. Furthermore, as the temperature was increased to 50 °C, the adsorption reduced, indicating that the process is less favourable at high temperatures. When an adsorbent is exposed to high agglomeration rates at high temperatures, it loses a lot of sorption effectiveness [57]. A similar finding was stated by Chaudhry, et al. [58] for adsorptive removal of Zn (II) and Pb (II) from water by using manganese oxide-coated sand adsorbent.

### 3.3. Removal of Zinc under Optimum Conditions

The adsorption procedure was performed at optimum conditions obtained in previous experiments. The D-WMR sample was added to a 100 mL solution containing 400 mg/L Zn^2+^ concentrations and agitated for three hours at 125 rpm. The highest adsorption capacity under optimal conditions was 25.0 mg/g. This is the moment at which the treatment becomes stable and no additional adsorption may occur due to the saturation of the surface area. Table 2 demonstrates that D-WMR has the highest adsorption capacity when compared to the other adsorbents in the literature.

**Figure 5:**
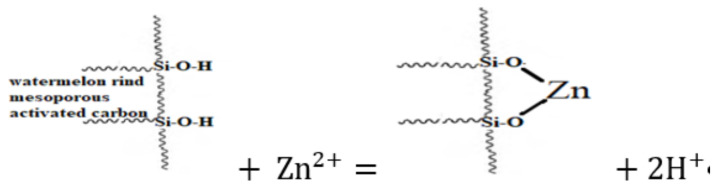
Zn^2+^ adsorption mechanism observed by D-WMR.

As a result, the adsorption process was completed at optimum conditions, and further treatment will not considerably improve Zn^2+^ removal. Furthermore, one of the Zn^2+^ adsorption mechanisms observed by D-WMR is ion exchange (reaction and fixation). The interaction between Zn^2+^ and exchangeable cation on the surface of mesoporous silica from dried watermelon rind may be represented as follows in Figure 5

### 3.4. Thermodynamic Model

#### 3.4.1. Principles of the Adsorption Thermodynamic Model

To assess the feasibility and nature of the adsorption process, thermodynamic parameters such as Gibbs free energy change (ΔG°), enthalpy change (ΔH°), and entropy change (ΔS°) were determined. At 20, 30, 35, and 40 °C (293.14, 303.15, 308.15 and 313.15 Kelvin), the thermodynamics of Zn^2+^ adsorption from respective solutions onto D-WMR with optimum parameters were investigated. As seen in the following equation, the change in free energy is related to the thermodynamic equilibrium constant, Kd [63]:

AG° = −RTLn Kd(8)

(9) $$\mathrm{Kd}=\frac{q_{e}}{C_{e}}$$

where:

Kd: The thermodynamic equilibrium constant (L g^−1^),

$q_{e}$: Adsorption capacity at equilibrium (mg g^−1^)

$C_{e}$: The concentration of Zn^2+^ ions in solution at equilibrium (mg L^−1^)

T: The absolute temperature in Kelvin,

R: The universal gas constant (8.314 J/mol K)

To make Kd dimensionless, equation 10 was adjusted as follows [64]:

(10) $$\mathrm{Kd}=\frac{q_{e}}{C_{e}}*\;\frac{M}{V}$$

where:

V: the volume of synthesized wastewater in a litter (L)

M: the mass of adsorbent of D-WMR in gram (g)

Another way to make Kd dimensionless is to use the following equation [65]:

(11) $$\mathrm{Kd}=\frac{C_{s}}{C_{e}}=\frac{C_{i}-C_{e}}{C_{e}}$$

where:

Kd: The thermodynamic equilibrium constant (dimensionless)

$C_{s}$: The amount of Zn^2+^ ions adsorbed (mg/L)

$C_{e}$: The Zn^2+^ ions concentration in the solution after equilibrium (mg/L)

$C_{i}$: The initial Zn^2+^ ions concentration in the solution (mg/L)

A proper equilibrium constant (Kd) value cannot be obtained from Equations (10) and (11) because Kd is not equilibrium constant, as reported in the literature [64,66,67]. The proper Kd calculation is provided in the section below.

#### 3.4.2. Equilibrium Constant Derived from the Langmuir Constant ($K_{L}$)

Isotherm studies were used to develop the Langmuir equation. The following reaction can be used to illustrate the connection between empty surface sites on an adsorbent’s surface (Sv; mmol/m^2^), adsorbate species in solution (A; mmol), and adsorbate species bound to surface sites (SA; mmol/m^2^).

(12) $$S_{V}+A⇌SA\;$$

The reaction has a constant Gibbs energy change (ΔG° J/mol) at all sites, according to the Langmuir formula, thus the thermodynamic equilibrium constant (Kd) is dimensionless. The major issue is that the Langmuir constant KL has dimensional units of L/mmol or L/mg, but the equilibrium constant Kd does not (without units). As a result, as numerous researchers have pointed out, using $K_{L}\;$(L/mmol or L/mg) directly in the determination of thermodynamic parameters gives incorrect results [67]. Several approaches have been suggested for resolving this unit issue. The equilibrium constant Kd may be easily derived as a dimensionless constant depending on the units of $K_{L}$. Kd may be easily calculated as a dimensionless parameter by multiplying $K_{L}\;$ by 55.5 and then by 1000 (Equation (13)) when adsorption research is done in an aqueous solution and $K_{L}$ has units of L/mmol [66].

(13) $$K_{d}=55.5\times 1000\times K_{L}$$

(14) $$\Delta \Delta G{}^{\circ}=-RTln\left(55.5\times 1000\times K_{L}\right)$$

(15) $$\ln\left(55.5\times 1000\times K_{L}\right)=\frac{-\Delta H{}^{\circ}}{R}\times \frac{1}{T}\times \frac{\Delta S{}^{\circ}}{R}$$

The number of moles of pure water per liter is 55.5 (1000 g/L divided by 18 g/mol), and the phrase 55.5 × 1000 × KL is dimensionless.

Milonjić [68] claimed that multiplying $K_{L}$ by 10^6^ (Equation (16)) yielded KL as a dimensionless parameter in the case of $K_{L}$ with units specified in L/mg. where 10^6^ is the density of the solution (assuming the density of pure water is 1.0 g/mL) and 10^6^ $K_{L}$ is a dimensionless quantity.

(16) $$K_{d}=10^{6}K_{L}$$

(17) $$\Delta G{}^{\circ}=-RTln\left(10^{6}K_{L}\right)$$

(18) $$\ln\left(10^{6}K_{L}\right)=\frac{-\Delta H{}^{\circ}}{R}\times \frac{1}{T}\times \frac{\Delta S{}^{\circ}}{R}$$

However, Zhou and Zhou [69] suggested that Kd might be derived as a dimensionless parameter by multiplying $K_{L}$ by the adsorbate’s molecular weight (Mw; g/mol), by 1000, and then by 55.5 (Equation (19)). The phrase Mw 55.5 1000$\;K_{L}$ is dimensionless.

(19) $$K_{d}=M_{w}\times 55.5\times 1000\times K_{L}$$

(20) $$\Delta G{}^{\circ}=-RTln\left(M_{w}\times 55.5\times 1000\times K_{L}\right)$$

(21) $$\ln\left(M_{w}\times 55.5\times 1000\times K_{L}\right)=\frac{-\Delta H{}^{\circ}}{R}\times \frac{1}{T}\times \frac{\Delta S{}^{\circ}}{R}$$

The Equations (16)–(21) can be used to determine the values of the parameters ΔG°, ΔH°, and ΔS°. Table 3 and Table 4 show the thermodynamic parameters estimated from Equations (16)–(21), respectively. Clearly, the Kd values were highly reliant on the Langmuir constant ($K_{L}$) and the methods that showed Kd as a dimensionless quantity. When the solution temperature was increased from 293.15 to 313.1 K, the values of ΔG° for Zn^2+^ adsorption decreased from −20.760 to −23.400 KJ/mol (Table 3) and from −23.901 to −26.756 KJ/mol (Table 4). The reduction of ΔG° values with increasing temperature indicates that zinc removal using D-WMR was more favorable and effective at higher temperatures. Furthermore, the negative values of ΔG° revealed that Zn^2+^ adsorption was a spontaneous process. Moreover, the values of ΔH° and ΔS° are determined by the intercept and slope of the plot lnKd versus 1/T. The ΔH° value (−0.195 KJ/mol) suggested that Zn^2+^ biosorption was a physical adsorption process. These findings also demonstrated that Zn^2+^ adsorption was controlled by an ion-exchange process [70]. In addition, the ΔS° for removing Zn^2+^ was determined to be 13.328 and 24.041 kJ/mol ∗ K. Consequently, the positive ΔS° values confirmed the increased randomness at the solid-solution interface during the removal process [71,72]. The release of H+ ions from the adsorbent surface might explain the positive entropy changes (ΔS°) for Zn^2+^ adsorption [58]. It is possible to conclude that the difference between the thermodynamic parameters estimated from the equilibrium constants derived from Equations (18) and (21) is insignificant.

### 3.5. Isotherm Models

The nonlinearized and linearized Langmuir and Freundlich models are utilized to simulate the bioadsorption data of Zn^2+^ adsorbed onto D-WMR in this work. The nonlinear and linear isotherm equation parameters’ correlations were examined and discussed. The Langmuir models’ nonlinear and linear equations are as follows [34,63]:

Langmuir

Nonlinear:

(22) $$q_{e}=\frac{q_{max}\;K_{L}\;C_{e}}{1+K_{L}C_{e}}$$

Linear:

(23) $$\frac{C_{e}}{q_{e}}=\frac{1}{q_{max}}C_{e}+\frac{1}{q_{max}}\;K_{L}$$

The Freundlich adsorption isotherm was developed for the heterogeneous process, and it describes the principle of multilayer adsorption on the adsorbent surface. Plotting qe versus Ce yielded the parameters of the Freundlich nonlinear model. The linear Freunlich isotherm constant values were derived by plotting ln qe vs. ln Ce. Freundlich’s nonlinear and linear equations were expressed as follows (respectively):

Nonlinear:

(24) $$q_{e}=K_{F}\;C_{e}^{1/nF}$$

Linear:

(25) $$\ln(q_{e})=\frac{1}{n}\;\ln(C_{e})+\ln(K_{F})\;$$

where $C_{e}$ (mg/L), $q_{e}$ and $q_{max}$ (mg/g) are the final Zn^2+^ ion concentration after the removal process, the adsorption capacity of the adsorbent at equilibrium and maximum Zn^2+^ removal capacity of D-WMR adsorbent, respectively.$\;K_{F}$ and nF (mg/g (L/mg) 1/n) are the Freundlich model constants related to the adsorption capacity and intensity, $K_{L}$ (L mg_1) is the Langmuir equilibrium constant related to the adsorption energy.

As stated in Equation (26), the equilibrium parameter or separation factor ($R_{L}$) was utilized to establish the favourability of adsorption in a particular concentration range:

(26) $$R_{L}=\frac{1}{1+K_{L}C_{i}}$$

**Figure 6:**
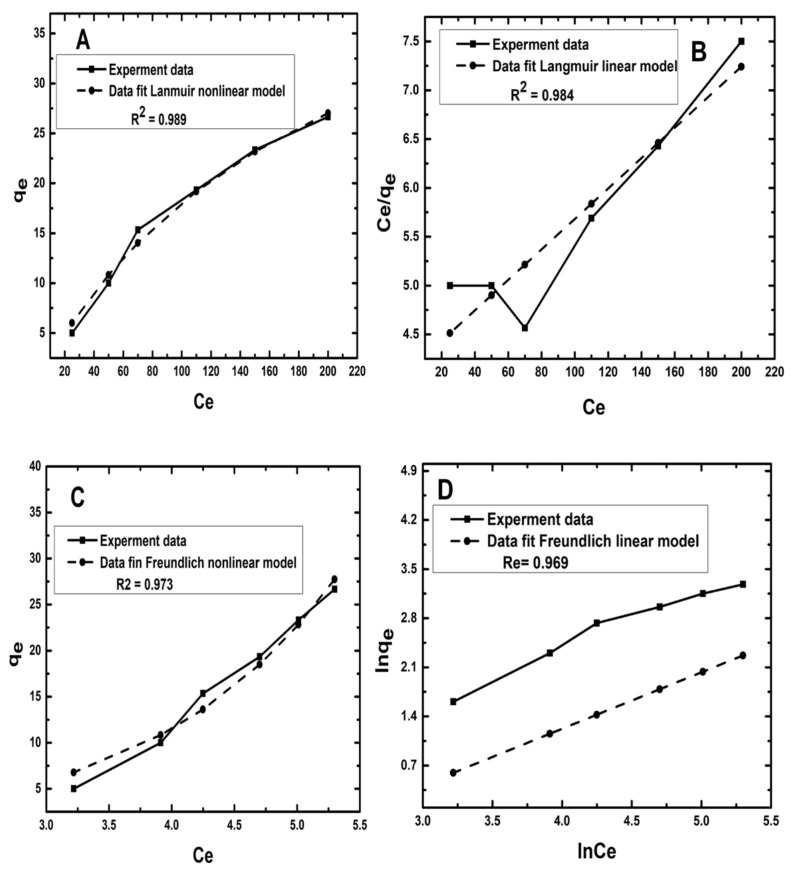
Nonlinear and linear isotherm models of Zn^2+^ adsorption from synthetic wastewater using D-WMR. (**A**,**B**): comparisons of the nonlinear and linear Langmuir isotherm models with experimental data. (**C**,**D**): Freundlich isotherm nonlinear and linear models of Zn2+ ions.

Figure 6A,B illustrate comparisons of the nonlinear and linear Langmuir isotherm models with experimental data (B). Moreover, Figure 6C,D present Freundlich isotherm nonlinear and linear models of Zn^2+^ ions, respectively. Table 4 shows the nonlinear and linear isotherm parameters as well as the correlation coefficients (R^2^). A high R^2^ demonstrated that the models accurately described the adsorption process. The Langmuir isotherm nonlinear model best predicted the adsorption equilibrium data according to the correlation coefficients (R^2^) analysis findings for each model in Table 4, despite the fact that the R^2^ value in the Langmuir nonlinear model for Zn^2+^ (0.989) is greater than that in the linear Langmuir model (0.984). The results of Langmuir isotherm models, on the other hand, reveal that Zn^2+^ adsorption occurred on a monolayer of D-WMR adsorbent. The Zn^2+^ analysis values obtained using Freundlich isotherm nonlinear and linear models were all lower than those estimated from Langmuir isotherm models due to the low R^2^ values. As a result, it can be concluded that the nonlinear Langmuir isotherm model is more suited to modeling Zn^2+^ ion adsorption on D-WMR. The 1/nF values derived using the Freundlich isotherm model, on the other hand, ranged from 0 to 1. These results suggested that Zn^2+^ ion adsorption on D-WMR was favourable [73].

### 3.6. Kinetics Models

The nonlinear and linear models of pseudo-first-order and pseudo-second-order kinetics were investigated in this work. However, both pseudo-first-order and pseudo-second-order kinetic models were examined, with the pseudo-second-order kinetic model expressing the experimental results better. The nonlinear and linear pseudo-first-order models were written as (respectively):

(27) $$q_{t}=q_{e}\left(1-e^{-K_{1\;}t}\right)$$

(28) $$\log\;(q_{e}-q_{t})=\log\;q_{e}-\frac{K_{1}}{2.303}t$$

The pseudo-second-order kinetic model was described primarily by Rout, et al. [74]. This kinetics model can be expressed by the following equation [75]:

(29) $$\frac{d_{qt}}{dt}=K_{2}\;\left(q_{e}-q_{t}\right)$$

Here $q_{t}$ symbolized the amount of Zn^2+^ adsorbed at time t and equilibrium (mg/g), and the rate constant for this reaction was symbolized by $K_{2}$ (g mg^−1^ min^−1^). The limits could be set into this equation, i.e., time t could range from t = 0 to t = t; qt also ranges from 0 to $q_{t}$, and then the Equation (29) changes to the following form:

(30) $$q_{t}=\frac{q_{e}^{2}K_{2}t}{q_{e}K_{2}t+1}$$

Now, the Equation (30) was linearized into the following forms [76].

(31) $$\frac{t}{q_{t}}=\frac{1}{q_{e}}\;t+\frac{1}{K_{2}\;qe^{2}}$$

**Figure 7:**
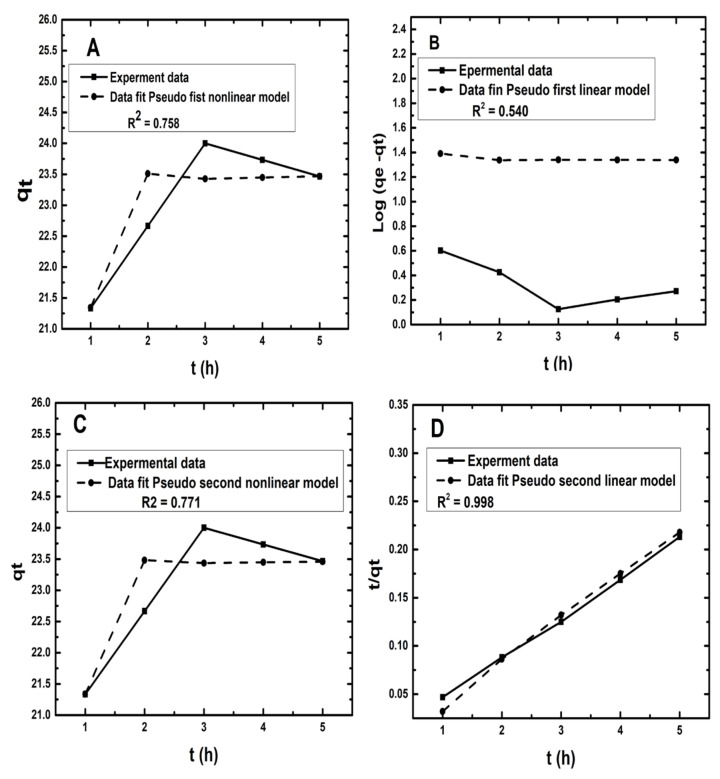
Nonlinear and linear kinetics models of Zn^2+^ adsorption from synthetic wastewater using D-WMR. (**A**,**C**): illustrate the findings of nonlinear models; (**B**,**D**): show the results of the linear models.

The slope and intercept of a plot of t/qt vs. t may be used to calculate $q_{e}$ and $K_{2}$. Table 5 summarizes the parameters of the pseudo-first and second nonlinear and linear models. According to the high R^2^ value, the experiments’ data were a better fit with pseudo-second-order kinetics than the pseudo-first-order kinetic model. The best fit of the pseudo-second-order kinetic model was achieved using correlation coefficients (R^2^). Because of the dependence on only one axis, the linear form was unsuitable for kinetic modeling [76,77]. Figure 7A,C illustrate the findings of nonlinear models, whereas Figure 7B,D show the results of the linear models. The difference in values derived by varying the linearized error structure may have an impact on the error distribution for the better or the worse [41]. As a consequence, the linear pseudo-second kinetics model with a high R^2^ value (0.998) accurately described the removal of Zn^2+^ using D-WNR. The use of a nonlinear equation avoids the need for any further translation to a linear form, reducing inaccuracy. Kinetic models were preferred over linear models because all of the parameters in this model remained constant during the kinetic modeling. This pseudo-second regression model proved more relevant and efficient for summarizing the complete experimental approach. Furthermore, the high correlation coefficient (R^2^) indicated that chemisorption was the best model for characterizing the adsorption mechanism [78].

### 3.7. Selecting the Optimum Isotherm Model Based on Error Functions Analysis

When choosing the best isotherm and kinetic models, the model with the highest coefficient of determination (R^2^) and the least amount of error function is usually recognized as the best fit model to the experimental data. However, because each model has at least one minimal error value, the coefficient of determination (R^2^) cannot only be assessed to find the optimal model. In this experiment, error values are crucial in choosing the optimal model. Due to its greatest R^2^ and least error values, the Langmuir (nonlinear) isotherm was given the first rank in Table 6’s results. Langmuir (linear) received the second rank, based on its R^2^ and least error values. The third is the Freundlich (linear) isotherm model, followed by the Freundlich (nonlinear) isotherm model, and finally the Freundlich (nonlinear) isotherm model. On the other hand, the best to worst order of kinetic models was the pseudo-second nonlinear model, followed by the pseudo-second linear model, pseudo-first nonlinear model, and pseudo-first linear model. Meanwhile, from Table 5, the Langmuir and Freundlich non-linear isotherm models and pseudo-first-order and pseudo second-order kinetic nonlinear models have lower value of MSE, RMSE, MAD and MAPE than their linear forms. Even though the R^2^ value of a linear pseudo-second-order model (0.998) is greater than the R^2^ value of a nonlinear pseudo-second-order model (0.771) (Table 5), the linear pseudo-second-order model has a higher error analysis value (Table 6). As a result, the nonlinear form of these models represents the adsorption process more precisely. Furthermore, all nonlinear regression error values (from Table 6) are lower than those derived by linear analysis, implying that the nonlinear fitting of experimental data into isotherm and kinetic models may have contributed to R^2^ value fluctuation and expected parameter variation. The non-linear has the lowest error levels when compared to linear, indicating that linearization changes the error distribution between the experimental and calculated values [79]. Because of their simplicity and utility in predicting the likely mechanism of adsorption data, linear forms were usually used to calculate isotherms and kinetics parameters. Nonlinear isotherm and kinetics models are converted to linear form, causing distortion in the linear least squares methodology [34]. It was not possible to verify the linearity of the data with these linear equations, because they presume that the data shown is linear in nature. Data points are assumed to be along a straight line, following Gaussian distribution, with standard error on the x-axis uniformly distributed [80]. For example, the isotherm and kinetic models did not follow this concept because their mechanisms were different and could be plotted using a nonlinear model instead. When using linear techniques, errors are distributed along the y-axis without taking into consideration the associated x-axis as well. Various categories of linear equations are affected by this assumption, resulting in different error analyses for the same experiment. Consequently, it was found that the linear technique was not suitable for the study of adsorption kinetics and could not be relied upon to provide a satisfactory conclusion of the whole modelling process. As a result of the above-mentioned limitations of linear equations, nonlinear equations were selected for describing the adsorption process [78].

## 4. Conclusions

The current study found that under optimal conditions, dried watermelon rind powder has a good capacity to extract Zn^2+^ ions from synthetic solution. The D-WMR was further examined using a scanning electron microscope (SEM) and X-ray fluorescence (XRF), with the findings confirming the removal of Zn^2+^ from synthetic wastewater. Moreover, at all temperatures, the negative values of ΔG° showed that the adsorption event happened favourably and spontaneously. The adsorption of Zn^2+^ ions onto D-WMR was more favourable at a higher temperature, as evidenced by the increasing negative values of ΔG° with the increasing of temperatures. The enhanced unpredictability and disorder at the solid/solution interface after the adsorption of Zn^2+^ ions onto D-WMR supports the positive value of ΔS°. The low adsorption enthalpy ΔH° indicates that the biosorption process was physical adsorption with just a weak contact between Zn^2+^ and the surface of the D-WMR. Additionally, the nonlinear Langmuir isotherm and nonlinear pseudo-second-order rate model described the experimental equilibrium data of Zn^2+^ ions onto D-WMR well. It is therefore more reasonable and accurate to use a nonlinear regression model to examine equilibrium adsorption data. According to the findings, D-WMR may be regarded as a natural and promising option for the removal of Zn^2+^ ions from wastewater.

## Figures and Tables

**Table 1:** Chemical composition of dried watermelon rind before and after adsorption of Zn^2+.^

Formula	Concentration before Zn^2+^ Adsorption	Concentration after Zn^2+^ Adsorption
ori-g	7	7
added-g	3	3
CO_2_	0.10%	0.10%
ZnO	-------	24.40%
Fe_2_O_3_	1.00%	1.34%
SiO_2_	83.70%	1.18%
SO_3_	8.32%	0.93%
Al_2_O_3_	1.61%	0.26%
Cr_2_O_3_	0	0.18%
P_2_O_5_	0.99%	0
CaO	2.18%	0.15%
K_2_O	0.87%	0
Cl	0.82%	0
MoO_3_	0.30%	0

**Table 2:** Adsorption of Zn^2+^ by D-WMR and other adsorbents from the literature.

Adsorbent	Uptake Capacity of Zn^2+^ mg/g	Reference
Oxidized jute	8.02	[59]
Neem bark	13.29	[60]
Clarified sludge	15.53	[60]
Black gram husk	13.45	[61]
NaOH-treated rice husk	20.08	[48]
Bentonite	10.75	[46]
Kaolin	3.70	[46]
Coir	8.6	[62]
Date stone	10.41	[51]
Modified Sugarcane bagasse.	9.23	[54]
Unmodified Sugarcane bagasse	12.25	[54]
D-WMR	25	This study

**Table 3:** Thermodynamic parameters of Zn^2+^ adsorption at different temperatures by using the Langmuir constant.

Temperature (Kelvin)	$K_{d}=10^{6}K_{L}$				
	Van’t Hoff Equation	Kd	ΔG◦ (kJ mol^−1^)	ΔH◦ (kJ mol^−1^)	ΔS◦ (J mol^−1^ K^−1^)
293.15	y = 0.0235x + 1.603 R^2^ = 0.9849	5000	−20.760	−0.195	13.328
303.15		6000	−21.927		
308.15		7000	−22.684		
313.15		8000	−23.400		

**Table 4:** Thermodynamic parameters calculated from the Langmuir constant.

Temperature (Kelvin)	$K_{d}=M_{w}\;\times \;55.5\;\times \;1000\;\times \;K_{L}$				
	Van’t Hoff Equation	Kd	ΔG◦ (kJ mol^−1^)	ΔH◦ (kJ mol^−1^)	ΔS◦ (J mol^−1^ K^−1^)
293.15	y = 0.0235x + 2.891 R^2^ = 0.9849	18143	−23.901	−0.195	24.041
303.15		21772	−25.176		
308.15		25400	−25.986		
313.15		29029	−26.756		

**Table 5:** Obtained parameters from linear and nonlinear isotherm and kinetic models.

Model	Parameters	Nonlinear	Linear
Langmuir	q_max_	53.918	64.103
	K_L_	0.005	0.0038
	R^2^	0.989	0.984
Freundlich	q_e_	0.679	0.805
	nF	0.762	0.762
	R^2^	0.973	0.9691
Pseudo-first-order	q_e_	23.679	3.898
	K_1_	2.317	0.204
	R^2^	0.758	0.540
Pseudo-second-order	q_e_	23.573	34.21
	K_2_	4.253	0.379
	R^2^	0.771	0.998

**Table 6:** Isotherm and kinetic error deviation data for adsorption of Zn^2+^ onto D-WMR using error functions.

Model		MSE	RMSE	MAD	MAPE
Langmuir	Nonlinear	0.598	0.774	0.634	0.066
	Linear	0.855	0.925	0.702	0.053
Feundlich	Nonlinear	1.486	1.219	1.125	0.109
	Linear	149	12.2	11.2	0.675
Pseudo-first-order	Nonlinear	0.187	0.432	0.286	0.012
	Linear	336	18.33	16.73	0.727
Pseudo-second-order	Nonlinear	0.177	0.421	0.279	0.012
	Linear	55.6	7.462	3.570	0.165

## Data Availability

Not applicable.
